# Eosinophilic Enteritis: A Delayed Diagnosis

**DOI:** 10.1177/2324709617734246

**Published:** 2017-10-03

**Authors:** Ankita Munjal, Abdulhameed Al-Sabban, Kathryn Bull-Henry

**Affiliations:** 1 Georgetown University Hospital, Washington, DC, USA

**Keywords:** eosinophilia, endoscopy, small bowel, pathology, abdominal pain

## Abstract

Eosinophilic gastrointestinal disorders are a rare and complex group of disorders that are characterized by eosinophilic infiltration of the gastrointestinal tract. Patients often present with a wide range of signs and symptoms as any length or layer of the GI tract can be involved such as mucosal, muscular, or serosal. As a part of the workup, patients frequently undergo computed tomography scans and multiple endoscopies before the diagnosis is finally made as was true in our case of a 59-year-old male patient presenting with 2 months of nausea, abdominal pain, and weight loss. He underwent esophagogastroduodenoscopies, colonoscopies, video capsule study, and balloon enteroscopy before the diagnosis was confirmed histologically. Endoscopic and radiographic findings can be variable and are usually unpredictable. The diagnosis is confirmed on histopathological examination of biopsies that must show >15-50 eosinophils/high-power field based on the location in the GI tract. In our patient, erythema, scalloping, whitish exudate, and patches of villous blunting were noted in the duodenum to proximal ileum endoscopically with >50 eosinophils/high-power field confirming the diagnosis of eosinophilic enteritis. This class of diseases is often found in patients with a history of allergic disorders suggestive of hypersensitivity in the etiology of the disease although our patient had no such known history. Elimination diets and steroids are the mainstay of therapy and often lead to complete resolution of symptoms as well as endoscopic and radiographic findings in up to 90% of patients as was seen in our patient, although some patients have a chronic remitting course.

## Introduction

Eosinophilic gastrointestinal disorders (EGIDs) are a rare and complex group of disorders that are characterized by eosinophilic infiltration of the gastrointestinal tract. Eosinophilic esophagitis (EoE) is limited to the esophagus while eosinophilic gastroenteritis (EGE) can involve any portion of the gastrointestinal (GI) tract. Patients often present with a wide range of signs and symptoms as any length or layer of the GI tract can be involved.^1^ As a part of the workup, patients frequently undergo computed tomography (CT) scans and multiple endoscopies before the diagnosis is finally made. Endoscopic findings are typically variable and often include mucosal erythema, edema, strictures, polypoid lesions, and ulcerations.^2^ Radiographic findings are also unpredictable and may include mural thickening, nodularity, luminal narrowing, and perienteric inflammation suggestive of an acute inflammatory condition.^2-4^ The diagnosis is confirmed on histopathological examination of biopsies that must show >15 to 50 eosinophils/high-power field based on the location in the GI tract. It is a disease that is often found in patients with a history of allergic disorders including asthma, eczema, seasonal allergies, and food allergies suggestive of hypersensitivity in the etiology of the disease.^5^ Elimination diets and steroids are the mainstay of therapy and often lead to complete resolution of symptoms, endoscopic and radiographic findings in up to 90% of patients, although some patients have a chronic remitting course.^2^

## Case Report

A 59-year-old Caucasian male presents with 2 months of intermittent nausea associated with abdominal pain, dysgeusia, weight loss, and diarrhea. He has no history of food, environmental, or drug allergies. The patient’s past medical history includes gastroesophageal reflux disease and colonic polyps found on screening colonoscopy. He had no significant family history including prior known allergic disorders. He denied any tobacco, alcohol, or illicit drug use. He was initially evaluated at an urgent care center and treated for oral candidiasis before referral to a gastroenterologist for persistent symptoms. As a part of the initial workup, he had an esophagogastroduodenoscopy (EGD) that revealed candidiasis in the esophagus, an ulcer in the stomach along with abnormal mucosa consistent with erosive gastritis, and duodenitis. Colonoscopy showed diffuse ulceration, granularity, erythema, and congestion with spontaneous bleeding throughout concerning for colitis. He had a CT of the abdomen/pelvis, which was unremarkable except for diverticula seen in the sigmoid colon and was started on prednisone 60 mg daily given concern for an unspecified colitis. After 1 month of treatment, the patient began feeling better with resolution of diarrhea, abdominal pain, and improvement in appetite. His steroids were tapered, and the patient presented with recurrence of symptoms a couple of months later.

**Figure 1.**
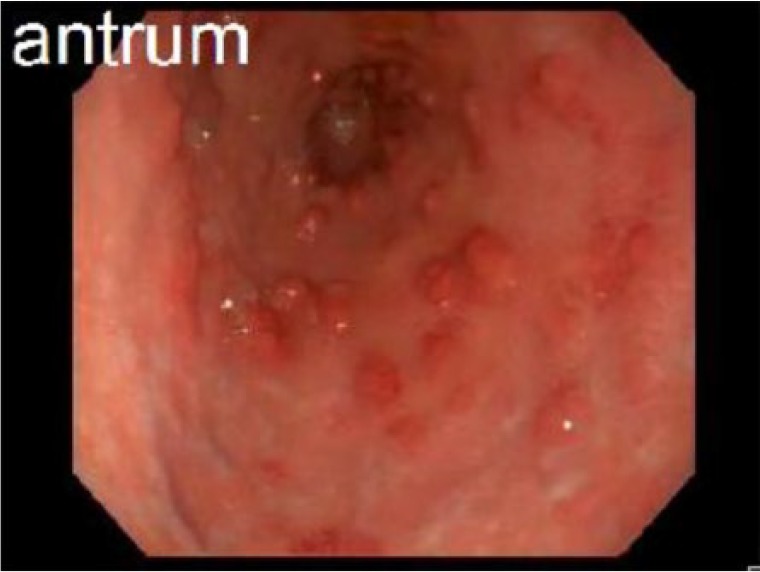
Endoscopy showing erythematous gastric nodules in the antrum of the stomach.

**Figure 2.**
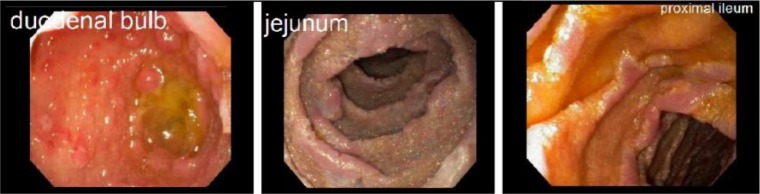
Endoscopy showing erythema, scalloping, and villous blunting throughout portions of gastrointestinal tract.

At that time, repeat EGD showed diffuse continuous granularity, congestion, and ulceration of mucosa without bleeding compatible with gastritis. Diffuse granularity and ulceration was also noted in the duodenum with pathology confirming villous blunting and reactive epithelial changes. He then underwent capsule study that showed erosive gastritis, duodenitis, and stigmata of bleeding. There were patchy mucosal changes in the proximal jejunum suggesting mild jejunitis as well. On balloon enteroscopy, erythematous gastric nodules and severe duodenal inflammation were noted as well as erythema, scalloping, whitish exudate, and patches of villous blunting with a flat pink appearance were seen throughout the jejunum until the proximal ileum as seen in Figures 1 and 2. Laboratory results were unremarkable without evidence of peripheral eosinophilia or hypoalbuminemia, and he had fungal cultures and *Helicobacter pylori* testing that were negative.

On histopathologic examination, biopsy of the duodenum, proximal ileum, and random jejunum showed small intestinal mucosa with moderate eosinophilia characterized as more than 50 per high-power field in the lamina propria, focal fibrosis, and villous blunting and atrophy confirming the diagnosis of eosinophilic enteritis. Immunohistochemistry was performed for CD117, IgG4, CD3, and tryptase to rule out a mast cell disorder or increased intraepithelial T cells. Mast cells were noted to be mildly increased but without atypia. No dysplasia, neutrophilic cyptitis, or granulomas were identified. He was continued on oral prednisone at discharge but subsequently lost to follow-up after establishing the underlying etiology of his symptoms.

## Discussion

EGID is a rare group of diseases characterized by eosinophilic infiltration of one or multiple layers of the bowel wall. It is a disease that can be seen from infancy to adulthood, but most commonly in the third to fifth decades.^5^ It has also been noted to be a disease most predominantly found in men as seen in our patient above.^6,7^ A disparity between incidence rates has been noted among types of EGID with EoE described more frequently in Western countries and EGE more commonly in Asian countries.^6^ Within EGE, most cases involve the stomach and proximal small bowel although, as in our case, jejunitis and ileitis are also described.^2^ The most common presenting symptoms of this disease include abdominal pain, nausea, vomiting, dysphagia, diarrhea, and even more serious manifestations such as intestinal obstruction or perforation.^5,8^ This is dependent on the layer of involvement in the gastrointestinal tract, such as mucosal, muscular, or serosal. In the mucosal form, which is the most common, patients often present with abdominal pain, vomiting, diarrhea, gastrointestinal bleeding, or malabsorption due to protein wasting, and it is associated with fold thickening, erythematous mucosa, and erosions on EGD.^2,9,10^ Disease involving the muscular layer leads to strictures, bowel wall thickening, and complications such as intestinal obstruction given decreased peristalsis and reduced distensibility, which is also seen on EGD.^2,9^ Serosal involvement often manifests with ascites and a high peripheral eosinophil count, which is unique to this subtype.^9^ CT imaging can also be useful in helping distinguish the depth of involvement based on specific radiographic findings, although they may be absent in at least 40% of patients.^2^ Endoscopic findings are also nonspecific and range from mucosal erythema, friability, nodularity, polyps, edema, ulceration, and fibrosis as well as complete loss of villi as seen in our patient.^2,11,12^ In EoE, in particular, whitish exudates and mucosal ridges may be seen on EGD, although interestingly, our patient had similar findings in the lower GI tract as well.^13,14^

As patients often present with a wide variety of signs and symptoms, this condition is often mistaken for similar disorders during the initial workup period, ultimately leading to a delay in diagnosis. There are several diagnostic criteria for the subtypes of EGID ranging from >15 to 50 eosinophils/high-power field in the lamina propria on histologic examination from at least 6 different biopsy sites to reduce sampling error.^2^ For our patient who presented with EGE, the diagnostic criteria include gastric or duodenal biopsies with >20 eosinophils/high-power field, which was confirmed in this case.^2,15,16^ Increased mast cells can also be present in EGID as seen with our patient.^15,17,18^ Given the increasing association with atopy, patients often undergo allergy testing to food and environmental triggers as well.^2^ It is also important to exclude other etiologies such as drug reactions, malignancy, or parasitic infections in addition to other organ involvement prior to confirming the diagnosis of EGID.^2,19^ Laboratory and serum testing can also be nonspecific in this group of disorders. Peripheral eosinophilia is typically associated with all subtypes of EGID, but it can be absent in up to 20% to 23% of cases as demonstrated in our case.^2,15^ Hypoalbuminemia, fecal protein loss, increased erythrocyte sedimentation rate, and elevated immunoglobulin E have also been reported in EGID patients.^2,6,8,19^ Stool and parasite culture should also be completed as a part of the workup, especially in patients with a history of diarrhea.

The pathogenesis of EGID is still unclear, but as 50% to 75% of patients with this disorder also have a personal or family history of food, medication, or pollen allergies, it is likely in the spectrum of hypersensitivity reactions due to specific inflammatory mediators including cytokines and eotaxin that ultimately lead to recruitment of eosinophils.^5,6,17,20,21^ The pathologic eosinophilic infiltration and degranulation then causes epithelial cell necrosis and villous atrophy as visualized on endoscopy.^2,15^ It is thought that food allergens cross the intestinal mucosa and trigger an inflammatory response in this class of diseases.^22,23^ Interestingly, the racial disparities noted among types of EGID are present in other immune and allergic disorders as well.^6^

EGID is treated with symptomatic management and reassurance in mild forms and restrictive diets, low-dose oral steroid therapy, and topical steroid therapy in more severe forms with variable responses.^2,5,24^ Often patients have complete resolution of symptoms and associated endoscopic findings after treatment is initiated. The clinical course of EGID, though, is variable based on subtype with quality of life more often being impaired in EGE given the extent of the GI tract involved and subsequent malnutrition.^6^ Appropriate duration of therapy is unknown as this is often a relapsing and remitting disease and treatment must be individualized to each patient.
